# High-Pressure Injection Damage Caused by a High-Pressure Washer

**DOI:** 10.7759/cureus.52498

**Published:** 2024-01-18

**Authors:** Noritaka Funato, Yuka Sugizaki, Yuhei Ri, Ryo Ichibayashi

**Affiliations:** 1 Department of Orthopedic Surgery, Toho University Medical Center, Sakura Hospital, Chiba, JPN; 2 Department of Urology, Toho University Medical Center, Sakura Hospital, Chiba, JPN; 3 Division of Emergency Medicine, Department of Internal Medicine, Toho University Medical Center, Sakura Hospital, Chiba, JPN

**Keywords:** diagnostic evaluation, mri, pressure gun injury, high-pressure injection, high-pressure injection injury

## Abstract

High-pressure injection injuries, caused by forcefully injecting liquids or gases into the body, present significant challenges in diagnosis and treatment. Complications such as infection and compartment syndrome can occur, leading to various outcomes, including the possibility of amputation.

Treatment approaches vary, with some cases undergoing surgery and others opting for conservative methods. However, due to the rarity of this injury, clear treatment guidelines are lacking. Consequently, there is insufficient data to establish specific guidelines, such as the duration of antibiotic treatment, necessity of surgery, and timing of rehabilitation intervention. While emergency surgery may be required, limited reports of detailed CT or MRI examinations being conducted before the surgical procedure are available.

This case report involves an initial assessment, including physical examination, X-rays, CT scans, and MRI, to determine whether surgical or conservative treatment is appropriate. The laboratory risk indicator for necrotizing fasciitis (LRINEC) score assists in evaluating the risk of infection, and MRI plays a crucial role in predicting complications.

## Introduction

High-pressure injection injuries occur when liquids or gases are forcefully injected into the body, leading to mechanical or chemical damage and the potential development of compartment syndrome [1-3]. Despite initially appearing as minor wounds, the injuries can be severe, and immediate treatment is crucial.

There are two approaches to treating this injury: surgery and conservative treatment. However, few case reports have been evaluated using CT and MRI, and there are no established guidelines regarding specific treatment strategies, so it is necessary to accumulate more cases.

We experienced a case of damage caused by a high-pressure injection that was evaluated using X-rays, CT, and MRI and cured with conservative treatment. We summarize its clinical features.

## Case presentation

**Figure 1:**
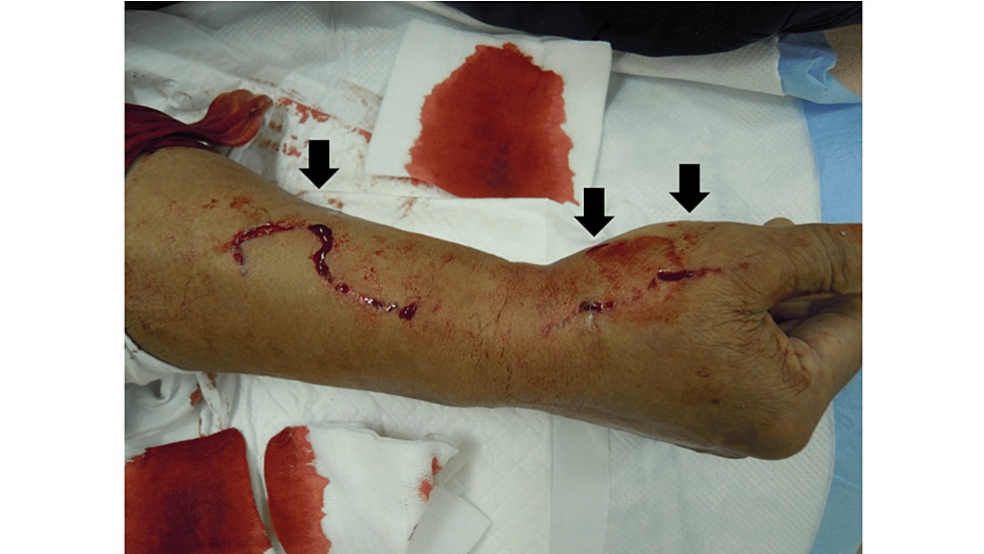
Wound findings on the day of injury The black arrows indicate open wounds. The entire forearm is swollen. Subcutaneous emphysema can be confirmed by palpation.

The patient was a 69-year-old man without a significant past or family history. He used well water and a commercial high-pressure washer to remove pipe rust. He was injured when the water pressure from the pressure washer accidentally hit his right forearm, and he came to the hospital on the day of the injury. When visiting the hospital, he was conscious with a blood pressure of 161/91, pulse rate of 65, and SpO2 100% (room air). His physical examination revealed three five-cm-long lacerations on the dorsum of his right hand and medial forearm (Figure 1).

**Figure 2:**
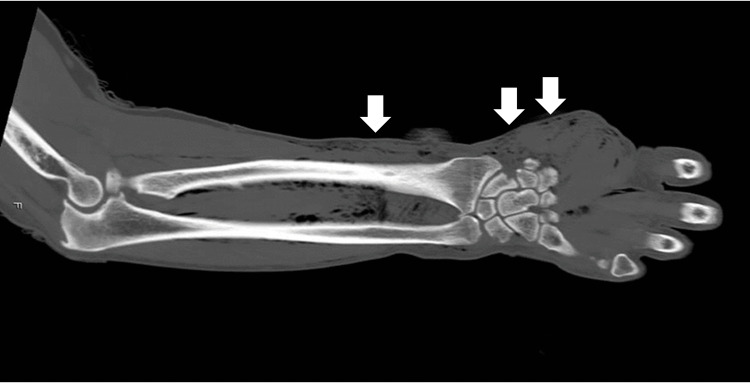
CT on the day of the injury White arrows indicate open wounds.

Persistent bleeding and swelling at the same site were noted. X-rays showed no fractures but subcutaneous emphysema. A CT scan of his right upper extremity revealed no fractures but emphysema in the soft tissue surrounding the forearm and upper arm between muscle compartments (Figure 2).

**Figure 3:**
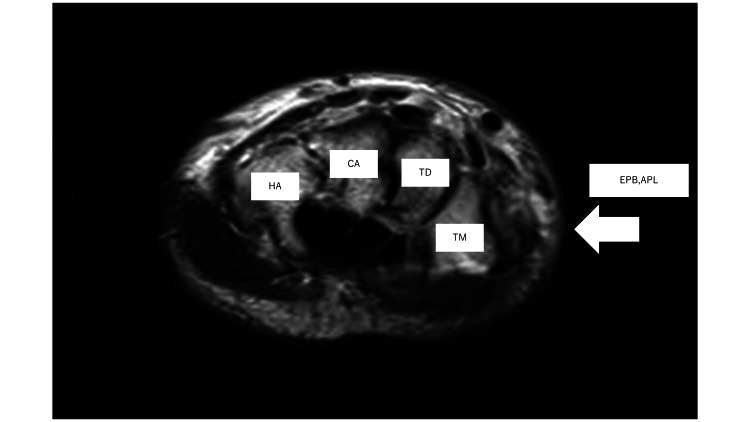
MRI of right hand joint (Short tau inversion recovery (STIR)) The day after the injury, swelling found in EPB (white arrow) EPB: Extensor pollicis brevis; APL: Abductor pollicis longus; Bone (TM: trapezium; TD: trapezoid; CA: capitate; HA: hamate)

Since he had no sensory or motor deficits, he was given antibiotics (ceftriaxone (CTRX)) and tetanus toxoid vaccine, and local wound care was performed on the wound. The wound was left open without sutures and protected with gauze. The wound was kept open because the well water used in the high-pressure washer might have been contaminated. The day after the injury, the redness and swelling at the injured site increased, so an X-ray examination and MRI of the same area were performed. MRI revealed edematous changes in the extensor pollicis brevis tendon, abductor pollicis longus tendon, flexor forearm muscles, adductor pollicis, and vermiformis (Figure 3).

**Figure 4:**
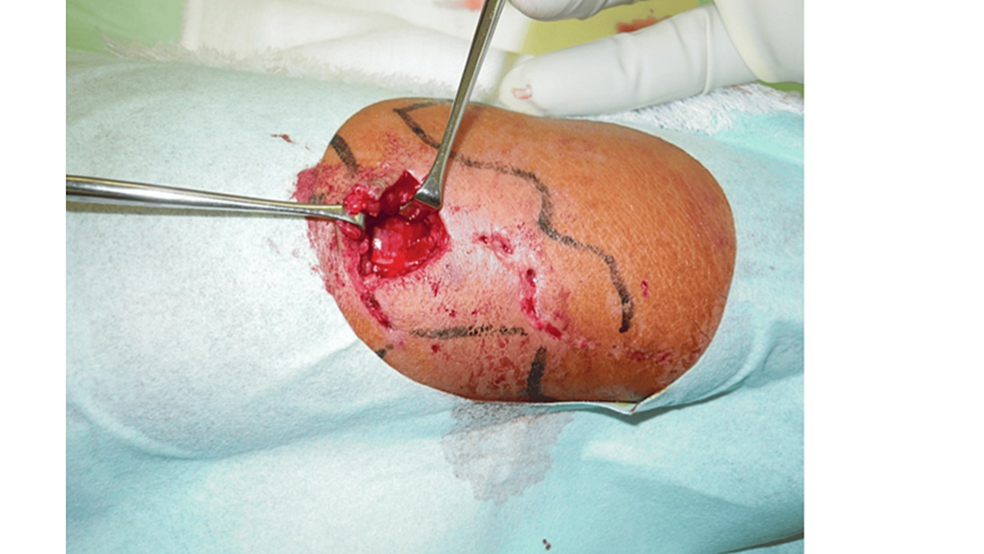
Test incision An approximately 2 cm test incision was made in the skin at the most painful part of the injury. No macroscopically poor color tone was observed in the fascia or muscle layer.

The laboratory risk indicator for necrotizing fasciitis (LRINEC) score was 0 on the day of injury but rose to 6 on the second day. The LRINEC score is an auxiliary diagnostic tool for diagnosing necrotizing fasciitis. The items used are blood tests such as C-reactive protein, white blood cells, hemoglobin, sodium, glucose, and creatinine. Necrotizing fasciitis is suspected when the LRINEC score is ≥6 points. For this reason, a test incision was made at the site of the most muscular local symptoms. He did not observe macroscopic discoloration within the fascia or muscle layer (Figure 4). He had a Penrose drain placed at the site of the test incision. He removed it two days later after confirming that the skin inflammation had not spread. At the same time, blood tests showed an inflammatory reaction, and he was given intravenous antibiotics for three days. After confirming no worsening of the inflammatory response, antibiotics were changed to oral administration. Oral antibiotics were administered for 15 days until the skin in the injured area improved.

On the sixth day after the injury, the overall pain over the site of the injury decreased, but the pain extended to the thumb became noticeable. On the ninth day of the injury, the pain persisted when extending and abducting the thumb, and Eichhoff's test was positive. Eichoff's test is a special examination technique used to evaluate stress in the abductor pollicis longus (APL) and extensor pollicis brevis (EPB). If positive, it indicates a disorder in the same area. On the 19th day after the injury, the skin at the injured site had almost improved, the pain in moving the thumb had improved, and Eichhoff's test had become negative. On the 29th day after the injury, it was confirmed that the symptoms had disappeared, and the hospital visit was concluded.

## Discussion

This case was caused by water pressure damage caused by a high-pressure washer using clean water. Evaluations using blood tests, CT, MRI, and test incisions revealed that the patient could heal with antibiotics and local wound treatment. During conservative, treatment the patient developed symptoms of pain in the movement of the thumb, but it was a muscle/tendon symptom that the initial MRI could explain.

Previously reported high-pressure water injection injuries included both surgical cases [4-7] and conservatively treated cases [2,7,8]. There have also been reports of cases where the injured area had to be amputated during surgery [4,9]. Regarding complications, local infections, sensory disturbances, muscle weakness, and neurological symptoms such as numbness have been reported. However, high-pressure injection injuries are rare injuries, and there are no reports of CT or MRI images being taken during water injuries. Factors that can cause complications include the degree of injury to nerves, blood vessels, and muscles caused by water pressure at the time of injury and the degree of water contamination. Adequate initial evaluation is essential for determining treatment strategy, but clear guidelines do not exist.

There's limited evidence in image evaluation due to the small number of cases. CT is more effective than X-rays at detecting foreign bodies and their three-dimensional location and can help plan surgery when foreign bodies are present in the body. For this reason, we recommend to always perform a CT scan if surgery is going to be undertaken. High-pressure washers can clean mud and dirt, sprinkle water at demolition sites, clean hallways, and tiles, remove dirt such as sand and dust before painting houses and condominiums, remove paint, and process machinery. If an MRI is performed with metal objects mixed into the body by a high-pressure washer, there is a risk of burns. For this reason, in the case of high-pressure jet damage caused by a high-pressure washer, it is essential to understand what was being cleaned. In this case, rust had been removed, so the possibility of rust getting into the wound could not be ruled out. Therefore, performing image evaluation using MRI after performing X-rays and CT was appropriate.

Emergency surgery is not considered appropriate unless there is muscle or blood vessel damage or foreign body contamination that X-rays or CT can confirm. There are different types of water: tap water, well water, and river water, and the state of water management differs depending on the region. When a high-pressure injection injury is caused by contaminated water, it is difficult to determine whether the wound should be opened [4,10]. However, since the distribution of the injected water can be confirmed with CT, if there is no distribution of water, there is no need to open and cleanse the wound. In addition, in injuries caused by high-pressure injections, if high water pressure is applied to the skin, resulting in a contusion, air and water may spread widely into the subcutaneous tissue via the soft tissue. In this case, air was also observed in the soft tissue layer between the muscle compartments up to the upper arm, but there was almost no water distribution. For this reason, the open wounds on the injured forearm and wrist were not closed and managed only by local disinfection. In addition, by administering antibiotics, the wound recovered without infection. To evaluate the injured area, it is necessary to take X-rays and CT scans, if possible, to assess whether the injected material was mainly air or water.

On the other hand, MRI can help assess not only the extent of the injury but also the muscles and tendons. Muscle damage is expected to occur more quickly in open wounds, where the most pressure is applied. For this reason, immediately after the injury, if the muscles in the open area are observed, and there are no problems, MRI imaging may not be necessary. This case was evaluated using the LRINEC score on the day of the injury, and test incisions were not made in the muscle. [11]. On the second day of the injury, the patient scored 6, so an MRI was taken. Edema within the muscle layer was detected, so a test incision was made. Since the muscle tone was expected, the fascia was closed, and the wound healed with only disinfection. The area where edematous changes in the muscle layer were found on MRI caused pain when extending and abducting the thumb during recovery, causing movement disorders. Therefore, early MRI scans may help predict complications and allow appropriate therapeutic intervention.

## Conclusions

The number of cases of high-pressure injection injuries is small, and guidelines do not exist. We have experienced a case of high-pressure injection damage with water from pressure washers. In addition to local findings, CT and MRI are useful for evaluating the extent of injury when deciding on a treatment plan. MRI can identify muscle damage caused by high-pressure injection, making it helpful in predicting complications and sequelae. Therefore, combining these tests is useful for determining treatment strategies.
